# Telemedicine in Low- and Middle-Income Countries During the COVID-19 Pandemic: A Scoping Review

**DOI:** 10.3389/fpubh.2022.914423

**Published:** 2022-06-22

**Authors:** Kareem Mahmoud, Catalina Jaramillo, Sandra Barteit

**Affiliations:** Faculty of Medicine and University Hospital, Heidelberg Institute of Global Health (HIGH), Heidelberg University, Heidelberg, Germany

**Keywords:** global health, digital health, telemedicine, low and middle income countries, telemedicine—utilization, low resource

## Abstract

**Background:**

COVID-19 has impacted the capacity of healthcare systems worldwide, particularly in low- and middle-income countries (LMICs), which are already under strain due to population growth and insufficient resources. Since the COVID-19 pandemic's emergence, there has been an urgent need for a rapid and adequate reaction to the pandemic's disruption of healthcare systems. To this end, telemedicine has been shown in prior research to be a feasible approach. The overarching objective of this scoping review was to determine the extent and acceptance of telemedicine in healthcare in low- and middle-income countries (LMICs) during the COVID-19 pandemic.

**Methods:**

This scoping review followed PRISMA guidelines and Arksey and O'Malley's five-stage framework to identify available evidence. We systematically searched four academic databases for peer-reviewed literature published between January 2020 and April 2021: Medline, PubMed, Web of Science, and Scopus, as well as Google Scholar as a source for grey literature.

**Results:**

The search identified 54 articles with 45,843 participants, including 6,966 healthcare professionals and 36,877 healthcare users. We identified a range of reasons for introducing telemedicine in LMICs during COVID-19, most notably to maintain non-emergency healthcare, enhance access to healthcare providers, and reduce the risk of infection among health users and providers. Overall, healthcare providers and users have shown a high level of acceptance for telemedicine services. During the COVID-19 pandemic, telemedicine provided access to healthcare in the majority of included articles. Nonetheless, some challenges to accepting telemedicine as a method of healthcare delivery have been reported, including technological, regulatory, and economical challenges.

**Conclusion:**

Telemedicine was found to improve access to high-quality healthcare and decrease infection risk in LMICs during COVID-19. In general, infrastructure and regulatory barriers found to be the most significant barriers to wider telemedicine use, and should be considered when implementing telemedicine more broadly. There appears to be a need to prioritize patient data safety, as many healthcare practitioners utilized commercial apps and services as telemedicine systems. Additionally, it appears as though there is a need to increase capacity, skill, and transparency, as well as to educate patients about telemedicine.

## Introduction

Globally, the introduction and use of telemedicine and telecare platforms has significantly grown in the context of the coronavirus disease (COVID-19) pandemic. Using telemedicine to mitigate the spread of infectious diseases is not unprecedented. Telemedicine services have been successfully deployed in combatting previous infectious disease outbreaks in LMICs, including the severe acute respiratory syndrome (SARS) outbreak in 2003 and the middle east respiratory syndrome (MERS) outbreak in 2015 considerably improving the healthcare system's response (1). Between 2014 and 2016, telemedicine services were also employed to help contain the Ebola outbreak in Africa. The Ebola contact tracing mobile application (app) was used to remotely monitor and track Ebola patients to halt the virus's spread from infected to uninfected individuals (2).

Since the COVID-19 epidemic, Langone Health Center in New York City reported an increase in telemedicine visits from 102.4 to 801.6 per day between March and April 2020, implying a 683% increase in visits in less than a month (3). Telemedicine services have made significant contributions to strengthen health care delivery, including screening patients for COVID-19 symptoms and offering online medical and mental health consultations throughout the pandemic. Similarly, many LMICs, including India, Lebanon and China, have already implemented telemedicine platforms that were extensively used during the COVID-19 pandemic (4). However, in many LMICs, telemedicine implementation and integration into the existing healthcare system are challenging; and may be a result of the high initial costs associated with telemedicine technologies, technological requirements such as reliable internet connectivity, or coordination issues between various sectors and stakeholders such as health ministries, science and technology ministries, local governments, and community hospitals. Oftentimes, in LMICs, government approval is required for the use of telemedicine, including well-defined regulations, legislation, and funding to facilitate telemedicine development and implementation, which may delay the adoption of telemedicine in such contexts.

Nevertheless, adopting telemedicine into clinical practice in LMICs may be a means to lower costs and conserve resources in the long run, thereby alleviating the burden of out-of-pocket spending and boosting the population's access to affordable healthcare. Particularly given that out-of-pocket payments for healthcare services make for a considerable share of overall health spending in many LMICs that have no general health insurance available (5). Furthermore, COVID-19 has put further strain on the already frail healthcare systems in LMICs. To this purpose, telemedicine services may alleviate pressure on the healthcare system by saving time and resources and strengthening healthcare's efficiency and accessibility. Furthermore, telemedicine may facilitate social distancing and limit face-to-face encounters in hospitals and clinics, hence preventing the spread of infectious diseases such as COVID-19 by physical contact. Teletriage, a subtype of telemedicine, has been identified as “a crucial method for managing disease epidemics,” as patients can be triaged before they arrive at healthcare facilities (6). Furthermore, telemedicine may help with counseling in relation to the COVID-19 pandemic since specific advice can be given to patients, for example, dos and don'ts in COVID-19 prevention (7).

Prior research includes, for example, a scoping review conducted by Hoffer-Hawlik et al. (8), who investigated telemedicine interventions for blood pressure control in LMICs and found that blood pressure was significantly reduced in telemedicine interventions, although the magnitude of the impact was not always substantial. They concluded that telemedicine may be an effective technique for boosting access to care and enhancing outcomes for hypertension in LMICs, especially during events that limit access to in-person care, such as the COVID-19 pandemic. However, large-scale, high-quality clinical trials are necessary to establish the efficacy and utility of telemedicine in hypertension therapy. The objective of Anthony Jr.'s (9) rapid review was to give theoretical and practical evidence on the value of telemedicine and virtual care for the distant treatment of patients during the COVID-19 pandemic in all countries. The review found that telemedicine and virtual platforms have the potential to aid in the management of large-scale epidemics and emergencies in high-risk environments. Moreover, it found that telemedicine permits the examination of a patient's health, while also digitally educating individuals about physical examination changes and symptoms that should provoke a dialogue with their doctors. In another literature search, Giacalone et al. (10) examined papers addressing the use of telehealth/telemedicine in the COVID-19 environment. They have discovered that the broad adoption of telemedicine services faces a number of obstacles that are mostly bureaucratic and regulatory in nature. In addition, it is crucial to make healthcare professionals and providers aware of this tool's limitations in order to prevent potential situations of carelessness. Prior to their acceptance, patients will need to be made aware of and trained on the usage of this new treatment method. In the present social and economic context, it is vital to establish a telehealth model that improves patients' quality of life and promotes the efficiency and continuity of healthcare. Bokolo (11) investigated how telemedicine and virtual software platforms can be used to treat outpatients during and after the COVID-19 pandemic, what telemedicine and virtual software platforms were used during and after the pandemic, and what factors influenced telemedicine and virtual software platform adoption. Overall, the analysis discovered that by remotely treating patients during and after the COVID-19 pandemic, telemedicine and virtual software are capable of reducing emergency department visits, protecting healthcare resources, and slowing the spread of COVID-19.

However, few insights are available on the barriers and facilitators of telemedicine for LMICs. Therefore, the main objective of this study was to map the existing literature on telemedicine to understand its scope and extent in LMICs during the COVID-19 pandemic. Additionally, the objectives were to determine the challenges and facilitators to telemedicine services in such contexts, as well as to determine how telemedicine may aid in the prevention of the spread of infectious diseases such as COVID-19 and to identify current research gaps. Furthermore, we aimed to focus on the following secondary research questions:

1. How were studies conducted in LMICs, what were specific study characteristics? What were technical modalities, including mode of communication (synchronous/asynchronous), platforms used? How was the telemedicine solution used in the clinical practice?
2. What were reported needs and reasons for employing telemedicine, particularly during the COVID-19 pandemic?
3. What were reported facilitators and barriers of using telemedicine in LMICs during the COVID-19 pandemic?

## Methodology

Due to the broad scope of the research question and the aim to include all types of studies, a scoping literature review seemed most suitable to generate insights to our research questions (12). The scoping review followed the methodological approach by Arksey and O'Malley (13) and Levac et al. (14), results are reported in line with the Preferred Reporting Items for Systematic Reviews and Meta-Analysis-Scoping Review (PRISMA-ScR) (15). Five iterative stages were involved in the review: (i) Identifying the research question, (ii) identifying relevant studies, (iii) selecting relevant studies, (iv) charting the data, and (v) summarizing results.

### Search Strategy

We searched four electronic databases as primary data sources to identify potentially relevant articles: Medline, PubMed, Web of Science, and Scopus. Due to the topic's recency, we included peer-reviewed preprints from medRxiv. Grey literature was searched using Google Scholar, and informed the introductory and discussion section (16).

We developed the search strategy from the three main concepts of “telemedicine,” “COVID-19,” and “low- and middle-income countries.” Based on test searches, we selected synonyms, Medical Subject heading (MeSH) terms, and additional keywords and altered the final search string to match the syntax requirements of each database (see Supplementary Material 1 for detailed search strings and search queries for the respective databases).

The World Health Organization defines telemedicine as “the delivery of health care services, where distance is a critical factor, by all health care professionals using information and communication technologies for the exchange of valid information for diagnosis, treatment, and prevention of disease and injuries, research and evaluation, and continuing education of health care providers, all in the interests of advancing the health of individuals and communities” (17). The WHO frequently refers to telemedicine as “healing at a distance” (17). Furthermore, conducting medical consultations over the phone or conferencing solutions (18), telemedicine platforms often allow for the transmission of supporting resources, such as radiological images and lab results, as well as text messages and email communications (19), whereby the mode of communication can be classified into two categories: synchronous and asynchronous communication. Synchronous communication comprises audio and video calls, whereas asynchronous communication consists of text messaging and email communications (20). We adhered to this scope of definition of telemedicine as part of this scoping review, which also includes disease and injury diagnosis, treatment, and prevention, as well as patient health status monitoring.

The search period was limited to 01.01.2020–30.04.2021 to encompass the first critical phase of the COVID-19 pandemic. The most recent search on the database Scopus was conducted on May 5th, 2021.

### Inclusion Criteria and Exclusion Criteria

We included studies conducted between January 2020 and April 2021, which focused solely on the development and usage of telemedicine platforms in LMICs, which includes all countries categorized by the World Bank as low-income (LICs), lower-middle-income (LMIs), or upper-middle-income (UMICs) in May 2021 (2, 3). Since the focus of this review was on telemedicine and the effects of COVID-19, we limited the study period to the onset of the COVID-19 pandemic through the most recent search date capturing as much of the COVID-19 pandemic as possible. Peer-reviewed articles were included only if they were published in English, included any kind of medical intervention offered by hospitals, clinics and healthcare providers via telemedicine services that were reachable by patients, as well as telemedicine services used for diagnosis, treatment and prevention of diseases and injuries via voice calls, video calls or text messaging services. The review included all aspects of healthcare, including mental health, dental, nursing, and rehabilitation. Due to the topic's recent nature and the need to identify research gaps, this scoping review included preprints and grey literature without regard for publication status.

### Study Selection and Eligibility

Two steps were used to identify relevant studies using Covidence software (21): (1) title and abstract screening and (2) full-text screening. To minimize bias, publications were examined individually by two reviewers (KM, CJ). Full-text screening was undertaken only by the first author, as is customary when scoping reviews are conducted (22). Our screening procedure was guided by defined inclusion and exclusion criteria developed using the population-exposure framework (PEO) framework (see Table 1 for details). Any disagreements were handled by mutual conversation. We contacted the first author *via* email if additional information was required for study selection and followed up twice after initial contact before dismissing the study.

**Table 1:** Inclusion and exclusion criteria based on the population-exposure-outcome framework.

	**Inclusion**	**Exclusion**
Population	• Adults and children seeking medical attention at hospitals, clinics, and healthcare providers that offer telemedicine services that are reachable by patients •Telemedicine services that are used for diagnosis, treatment and prevention of disease and injuries •Telemedicine services offered via video calls, voice calls or messaging services. •All medical fields, including mental health •Research conducted exclusively in a LMIC^*^	• Location not stated •Study population not specified •No patients as study participants •Research conducted in or about high-income countries •Telemedicine services used for research and evaluation and the continuing education of health care providers
Exposure	• Research primarily conducted about telemedicine	• Studies not focusing on telemedicine services
Outcome	• Studies reporting at least one use of the MOOC in at least one LMIC	• Studies in which the MOOC was only planned, not implemented
Time	• Published after 1 January 2020 and 31 April 2021	• Published before 1 January 2020 •Published after 31 April 2021
Study type	• Any primary, peer-reviewed research •Gray literature included •Full text available	• Secondary/synthesis research •Full text not available
Language	• English	• Languages other than English

* *LMICs as defined by the World Bank as of January 2021*.

### Synthesis of Results

While most of the extraction criteria were generated a priori and in accordance with the study objectives, some were revised throughout the extraction process to accommodate additional information. We extracted the Digital Object Identifier (DOI), title, author's name, year of publication and full text Uniform Resource Locator (URL), the country each study was conducted in, the World Bank Classification of these countries. The outcomes of each study were extracted as results and conclusions (see Supplementary Material 2 for complete data extraction template).

## Results

**Figure 1:**
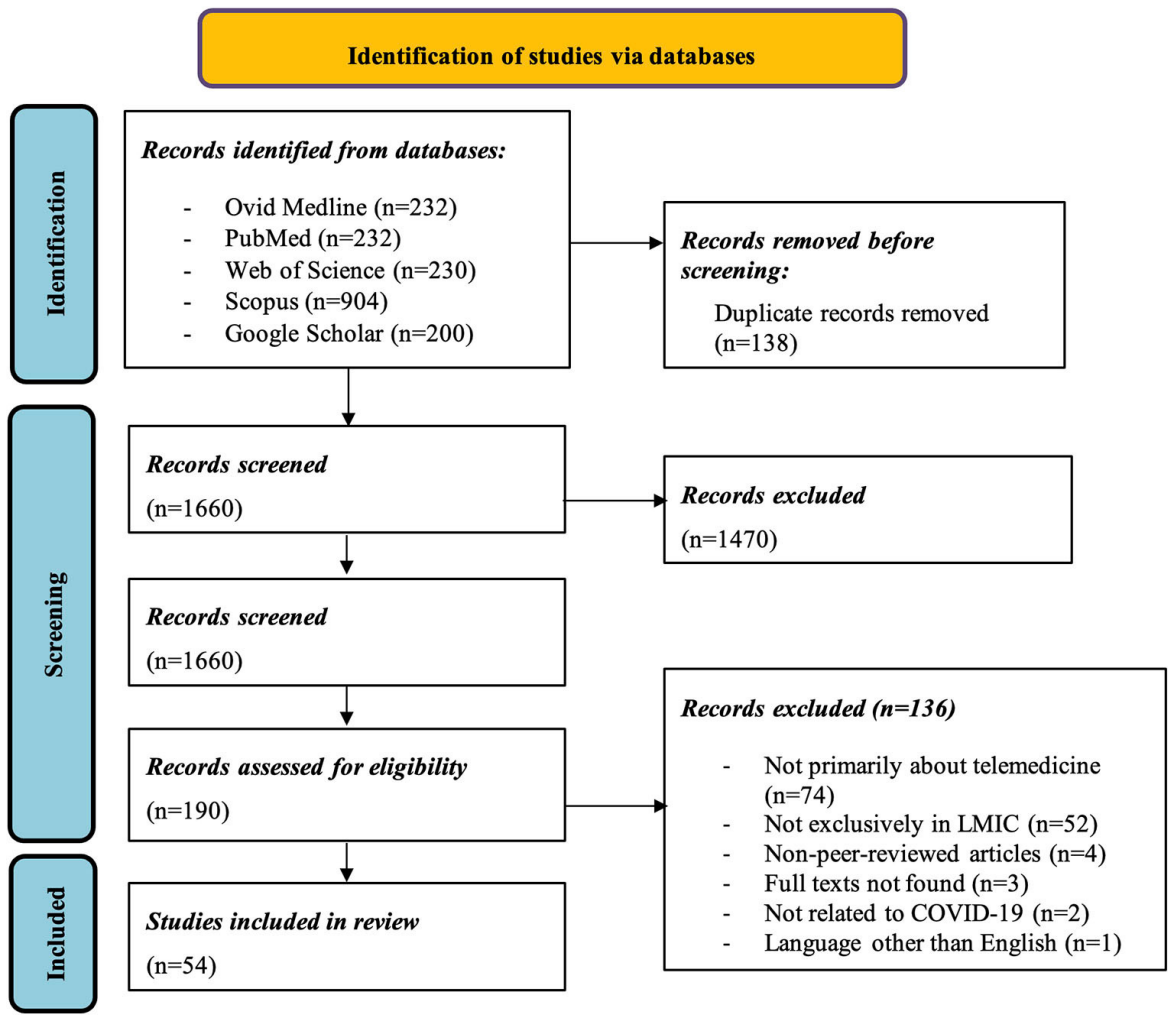
PRISMA flow chart.

The systematic search of the databases returned 1,798 articles of which we included 54 articles. The main reasons for exclusion were: not exclusively based in low- and middle-income countries, not peer-reviewed, no full text available, and no relation to COVID-19 (see Figure 1 for details, PRISMA flow chart).

### Characteristics of Included Studies

This review included 45,843 participants, comprising 6,966 healthcare professionals and 36,877 healthcare users (see Table 2 for details on characteristics of included studies). Almost half of the articles (*n* = 23, 42.6%) included healthcare users as study participants, healthcare providers were covered in a smaller number of articles (*n* = 5, 9.3%). Many articles focused on telemedicine in the context of a specific medical discipline, whereby the most covered disciplines were rehabilitation (*n* = 5, 9.3%), mental health (*n* = 4, 7.4%), neurology (*n* = 4, 7.4%). and dermatology (*n* = 3, 5.6%).

**Table 2:** Key characteristics of included studies.

**Characteristic**	**Number of studies (*n*)**	**Percentage (%) of all studies**
**Year of publication**		
−2020	30	55.6%
−2021	24	44.6%
**Country**		
- India	13	24.1%
- China	10	18.6%
- Brazil	7	13.0%
- Pakistan	3	5.6%
- Turkey	2	3.7%
- Libya	2	3.7%
- Egypt	2	3.7%
- Mexico	2	3.7%
- The Philippines	2	3.7%
- Lebanon	1	1.9%
- Peru	1	1.9%
- Colombia	1	1.9%
- Kosovo	1	1.9%
- Malawi	1	1.9%
- North Macedonia	1	1.9%
- Ecuador	1	1.9%
- Iran	1	1.9%
- Sub-Saharan Africa	1	1.9%
**World Bank classification**		
- Low-income economies	1	1.9%
- Lower-middle-income economies	21	38.9%
- Upper-middle-income economies	31	57.4%
- Mixed	1	1.9%
**International Bank for Reconstruction and Development (IBRD) countries**		
- Yes	49	90.7%
- No	4	7.4%
- Mixed	1	1.9%
**Urban/rural**		
- Urban	4	7.4%
- Rural	1	1.9%
- Urban and Rural	13	24.0%
- No mention	36	66.7%
**Study perspective**		
- Healthcare user only	23	42.6%
- Healthcare provider only	5	9.3%
- Healthcare system only	10	18.5%
- Healthcare user and healthcare provider	7	13.0%
- Healthcare user, healthcare provider and healthcare system	9	16.9%
**Medical specialty**		
- Multi-Specialty	22	38.6%
- Rehabilitation	5	9.3%
- Mental health	4	7.4%
- Neurology	4	7.4%
- Dermatology	3	5.5%
- Dentistry	2	3.7%
- Pediatrics	2	3.7%
- Ophthalmology	2	3.7%
- Diabetology	1	1.9%
- Emergency Medicine	1	1.9%
- Immunology	1	1.9%
- ICU	1	1.9%
- Rheumatology	1	1.9%
- Urology	1	1.9%
- Vascular surgery	1	1.9%
- Virology/Sexology	1	1.9%

Most studies were conducted in middle-income countries, with a quarter of the included studies (*n* = 13, 24%) conducted in India, ten studies (18.5%) in China, seven studies (13.0%) in Brazil, and four studies (*n* = 4, 7.4%) in Turkey. Only one study (1.9%) was conducted in a low-income country.

### Telemedicine Characteristics

We categorized telemedicine characteristics based on themes that emerged throughout the data charting process (thematic analysis), as presented in the following.

#### Communication Modes

More than half of the included publications (*n* = 32, 59.3%) discussed the use of both synchronous and asynchronous communication methods in telemedicine. Asynchronous communication was used by about one-quarter of the included publications (*n* = 12, 22.2%), with only one study (1.9%) using asynchronous modes. The mode of communication was not specified in a high number of articles (*n* = 9, 16.7%).

#### Telemedicine Platforms Used

WhatsApp was the most often mentioned platform, appearing in almost a quarter of the articles (*n* = 15, 27.8%). Telephone conversations were reported the second most frequently (*n* = 10, 18.5%). Eight studies (14.8%) used specific telemedicine platforms designed by healthcare providers, seven studies (13%) reported the use of Zoom. Furthermore, email was used in some studies (*n* = 6, 11.1%), as well as “WeChat” (a Chinese multipurpose messaging and social media application; *n* = 5,% = 9.3) articles. A few studies (*n* = 3, 5.6%) reported using Google duo/hangouts, FaceTime, Skype, and SMS, as well as unspecified publicly available commercial smartphone applications. Microsoft teams were mentioned in (*n* = 2, 3.7%) of the articles. Each app was mentioned once: “Updocs”, “Vsee”, “Cisco Webex meeting”, “Facebook Messenger”, “Viber” and fax.

#### Telemedicine Applications

We have categorized and described in more detail how telemedicine services were employed in clinical practice in the following (see Table 3 for detailed information).

**Table 3:** Overview of telemedicine applications.

**Application**	**No. studies (*n*)**	**Studies (%)**
Medical consultation	23	42.6%
Patient follow-up	18	33.3%
Specialist consultations	11	20.3%
Laboratory tests, drug prescription and delivery	11	20.3%
Teletriage and screening	6	11.1%
Counseling and telerehabilitation	5	9.3%
Other telemedicine applications	7	13.0%

#### Medical Consultations for the Purpose of Diagnosis

Participants in nearly half of the studies (*n* = 23, 42.6%) had a medical consultation with a healthcare practitioner for diagnosis. For example, Hoagland et al. (23) provided 564 HIV-positive research participants HIV self-test kits, followed by telemedicine consultations, in order to get pre-exposure prophylaxis (PrEP). Montenegro et al. (24) offered palliative consultations to some of the 273 cancer patients in their study, while Morgenstern-Kaplan et al. (25) offered almost 2,500 free video consultations to 1,545 pediatric patients. Mostafa and Hegazy used email for asynchronous teleconsultations with 62 dermatological patients, Shalash et al. provided for 19 patients a neurological evaluation during a virtual visit, including motor and non-motor examinations (26, 27).

#### Patient Follow-Up

Follow-ups with chronic condition patients were the second most reported telemedicine application. Alessi et al. (28) conducted a study in which 91 study participants with a prior diagnosis of type 2 diabetes were followed up via phone consultations lasting 5–10 mins each for 16 weeks. Participants in the study were unable to access outpatient clinics as they were closed due to COVID-19 restrictions. According to Montenegro et al. (24), the 273 patients who took part in their study were provided follow-up visits to answer questions and manage comorbidities.

#### Specialist Consultations and Health Information Exchange Between Healthcare Providers

A number of studies (*n* = 11, 20.4%) used telemedicine to connect and transfer health information between healthcare practitioners and healthcare professionals, to conduct specialist consultations, and to acquire a second opinion. Hong et al. (29) reported that specialists and consultants were able to remotely assess patients in the presence of a local primary healthcare provider using remote Computed Tomography (CT) devices, exchanging patient health information, medical records, images, and laboratory results in a synchronous manner. Sahu et al. (30) focused their study on 68 participants receiving treatment for Substance and Alcohol Use Disorder (SAUD) in India who were unable to access healthcare facilities due to the COVID-19 lockdown via an asynchronous email communication between primary healthcare practitioners and psychiatrists.

#### Laboratory Tests, Drug Prescription and Delivery

Telemedicine was utilized in 11 studies (*n* = 11, 20.4%) for laboratory testing, drug prescription, and/or delivery. Hoagland et al. (23) lowered the number of visits for PrEP refills on study participants to every 120 days, rather than at least three times per 90 days, which resulted in a considerable reduction in time spent in the health care facility. Montenegro et al. (24) conducted a study in which 273 study participants received laboratory testing services at home, and their needed medicines were delivered directly to their door. Similarly, Shenoy et al. (31) showed that telemedicine may be utilized to diagnose and treat a rheumatic patient by requiring participants to upload previous lab results using the commercial messaging service WhatsApp prior to participating in a video conference with their healthcare professionals. Following the consultation, the healthcare provider prepared the prescription, which was quickly packed and delivered by a local pharmacy courier.

#### Teletriage, Screening, and Pandemic Management

Some studies (*n* = 6, 11.1%) reported on the use of telemedicine in teletriage, screening, and pandemic management. Matheus et al. (32) identified telemedicine as playing a significant role in alleviating the effect of the COVID-19 pandemic on the healthcare system. Also, Turan and Utlu (33) reported that telemedicine was effective in triaging and screening 468 dermatological patients, resulting in a significant decrease in in-person visits to the healthcare facility.

#### Counseling and Telerehabilitation

A few studies' (*n* = 5, 9.3%) reported on the use of telemedicine as a tool for counseling and rehabilitation. Samadi et al. (34), for example, focused on a daycare center that specialized in children with autism spectrum disorder (ASD) and provided online counseling in the form of individual or group sessions to 336 carers of children with ASD, utilizing both synchronous and asynchronous approaches.

#### Other Telemedicine Applications

Perez-Noboa et al. (35) reported on telemedicine for continuous vital sign monitoring, which used telemedicine in conjunction with body sensors to provide healthcare providers with continuous updates on the patient's health status. Caetano et al. (6) identified telemedicine as a component of “online/virtual hospitals,” stating that “virtual clinics can be assembled using telemedicine consultations, including imaging tests (e.g., chest x-ray and/or chest computerized tomography—CT, relevant for assessing pulmonary involvement from the coronavirus), uploaded from peripheral sites and interpreted remotely,” ensuring that patients received clinical care while also minimizing physical crowding in hospital facilities.

### Needs/Reasons for Employing Telemedicine

The majority of studies (*n* = 47, 87%) discussed the importance and necessity of implementing telemedicine services (for an overview, see Table 4), providing a variety of arguments and motivations, whereby three key themes emerged: (i) inaccessibility of health care services, (ii) high risk of infection, and (iii) low resource setting.

**Table 4:** Overview of needs and reasons for employing telemedicine.

**Needs/reasons for employing telemedicine**	**No. studies (*n*)**	**Studies (%)**
Restriction or disruption of routine healthcare due to COVID-19 measures	31	57.4%
Lack of physicians	16	29.6%
Socio-economic reasons	4	7.4%
Protection of healthcare users/patients	13	24.1%
Protection of healthcare providers	9	16.7%
Pressure-relief of overburdened healthcare system	9	16.7%
Shortages in personal protective equipment (PPE)	4	7.1%

Over half of included studies (*n* = 31, 57.4%) discussed the need of telemedicine in resuming the provision of healthcare services that had been halted owing to lockdown measures, movement restrictions, and social distancing policies. Several articles (*n* = 16, 29.6%) recognized telemedicine as a promising option to compensate for doctors' limited availability (6, 7, 31, 35–47). Also, several others (*n* = 13, 24.0%) identified telemedicine as a preventive method for reducing physical interactions and hence lowering the risk of patients contracting COVID-19 (48–50) Some studies (*n* = 9, 16.7%) reported lowering the risk for healthcare workers (6, 24, 26, 35, 44, 48, 50, 51), others (*n* = 4, 7.4%) emphasized telemedicine's need to address disparities in healthcare worker distribution and high healthcare costs (40, 42, 47, 51, 52). Telemedicine was an important aspect to avoid a shortage of personal protective equipment (PPE) in healthcare facilities (*n* = 4, 7.4%) (6, 11, 23, 25, 39, 41, 43).

### Reported Benefits of Telemedicine

Reported benefits were classified as benefits for (i) healthcare users, (ii) healthcare providers, (iii) healthcare system, and (iv) the environment (see Table 5 for an overview).

**Table 5:** Overview of reported benefits of using telemedicine.

**Reported benefits**	**No. studies (*n*)**	**Studies (%)**
**Healthcare users**		
- Increased accessibility to healthcare	22	44.4%
- Financial saving	10	18.5%
- Time saving	8	14.8%
- Reduced need for transportation	10	18.5%
- Enhanced health outcomes	9	16.7%
- Healthcare user satisfaction	3	5.6%
**Healthcare providers**		
- Financial saving	5	9.3%
- Time saving	9	16.7%
- Reduced need for transport	7	13.0%
- Healthcare provider satisfaction	3	5.6%
**Healthcare system**		
- Less overcrowding	4	7.4%
- Reduced risk of infection	17	31.5%
- Saving resources	11	30.4%
- Continuation of care	8	14.8%
- Digital health record	9	16.7%
**Environmental benefits**		
- Reduced traffic pollution	2	3.7%

#### Healthcare Users

Almost half of the studies (*n* = 24, 44.4%) reported that telemedicine's use increased healthcare users' access to healthcare professionals and specialists who were previously inaccessible due to the long travel distances or due to COVID-19-related restrictions (4, 6, 7, 23–26, 30, 35–42, 51, 53–59).

According to some studies (*n* = 10, 18.5%), telemedicine has resulted in lower healthcare costs for patients (4, 6, 7, 24, 29, 36, 37, 51, 54, 55), reduced time spent seeking medical advice (*n* = 8, 14.8%) (6, 7, 37–40, 54, 60), and less transportation to reach healthcare facilities (*n* = 10, 18.5%) (6, 7, 25, 27, 37, 41, 42, 54).

Telemedicine has shown in several studies (*n* = 9, 16.7%) to improve healthcare outcomes and the quality of care received (26, 27, 35, 42, 43, 47, 50, 52, 61, 62). A few articles (*n* = 3, 5.6%) have also indicated high satisfaction levels among healthcare users with telemedicine-assisted care (4, 23, 25). Additionally, these studies found that healthcare users were confident in their competence to use such tools to obtain health advice.

A few articles (*n* = 17, 31.5%) showed that telemedicine lowered the risk of infection by reducing the number of individuals in healthcare institutions who could spread COVID-19 or other infectious diseases (23–26, 29, 41, 43, 44, 48–52, 62–64). Telemedicine was found to enable the continuation of healthcare services in several studies (*n* = 8, 14.8%) that would have been restricted or disrupted otherwise due to COVID-19 restrictions (4, 7, 25, 28, 51, 57, 64, 65).

#### Healthcare Providers

A financial benefit was reported in a few studies (*n* = 5, 9.3%) including savings for the healthcare provider that would otherwise be spent by physically attending the healthcare facility (6, 24, 27, 53, 54). Also, time savings were reported (*n* = 9, 16.7%) (24– 27, 43, 46, 52, 60, 64), as well as significant reduction in transportation needs to reach healthcare facilities (*n* = 7, 13%) (6, 7, 27, 30, 37, 52, 54). Three studies (5.6%) reported healthcare providers to be quite satisfied and confident in adopting telemedicine services (4, 27, 30). Four studies (7.4%) concluded that telemedicine helped alleviate overcrowding in healthcare facilities by reducing the number of visitors required to be physically present (31, 44, 46, 50).

#### Healthcare System

Telemedicine saved healthcare systems resources in 11 studies (20.4%), including saving time and strategically deploying people to obtain the most time- and cost-effective results. Saving PPEs was also part of the healthcare system savings (23, 29, 36, 37, 40, 42, 57, 65–67). Some articles (*n* = 8, 14.8%) claimed that adopting telemedicine has resulted in the continuation of healthcare services that would otherwise be constrained or disrupted due to COVID-19 constraints and rules (4, 6, 33, 35, 36, 39, 41).

Nine studies (16.7%) observed improved health record management and access to health information for healthcare users (6, 24, 29, 30, 35, 36, 45, 60, 62). Additionally, telemedicine facilitated information sharing between healthcare users and clinicians and between doctors and other providers.

#### Environmental Benefits

A minority of studies (*n* = 2, 3.7%) outlined a positive effect of telemedicine on the environment by reducing the usage of vehicles such as cars, resulting in potentially lower emission levels (4, 35).

### Facilitators of Using Telemedicine in LMICs

We categorized facilitators as following: technological facilitators, regulatory facilitators, personal facilitators, and professional facilitators (see Table 6 for an overview).

**Table 6:** Overview of identified facilitators of telemedicine in LMICs.

**Reported facilitators**	**No. studies (*n*)**	**Studies (%)**
**Technological**		
- Availability of infrastructure	19	35.2%
- High mobile phone usage	15	27.8%
- High internet usage	6	11.1%
**Regulatory personal**	11	20.3%
- Positive attitude towards telemedicine	6	11.1%
- Support of family and caregivers	3	5.6%
**Professional**	3	5.6%

#### Technological Facilitators

Many studies (*n* = 19, 35.2%) reported the availability of infrastructure to support telemedicine's use, such as broadband cellular network technologies, simple mobile applications (4, 23–25, 27, 34–38, 45, 53, 55–57, 65, 66, 68), mobile phones (*n* = 15, 27.8%) (23, 26, 27, 29, 32, 35, 39, 45, 48, 52–55, 57, 66), as well as wider internet (*n* = 6, 11.1%) (36, 37, 39, 40, 65, 66).

#### Regulatory Facilitators

In response to the restrictions imposed by the COVID-19 pandemic and the limitation and disruption of provision of care in many healthcare facilities and departments, some countries such as Brazil, India and Lebanon have passed new legislations and regulations that enable, facilitate, and in some cases regulate the use of telemedicine as an alternative form for provision of healthcare (4, 6). Almost a quarter of included studies (*n* = 11, 20.3%) have reported that similar enabling legislation and laws have been set (4, 7, 29, 30, 32, 36, 38, 46, 59, 61, 66).

#### Personal Facilitators

A few studies (*n* = 6, 11.1%) examined positive attitudes toward telemedicine platforms used, including acceptability and satisfaction (25, 38, 53, 54, 62, 66). Three studies (*n* = 3, 5.6%) addressed the function of family and caregivers in facilitating telemedicine consultations (24, 34, 48).

#### Professional Facilitators

A small number of studies (*n* = 3, 5.6%) indicated that healthcare providers received specialized training on using telemedicine platforms and adhering to the protocols required for telemedicine consultations (43, 63, 68).

### Reported Barriers to Implementation of Telemedicine Services

Common themes relating to barriers of telemedicine have emerged (see Table 7 for details), which we categorized as (i) technological, (ii) regulatory, (iii) financial, (iv) related to healthcare users, (v) related to healthcare providers, (vi) quality of care, and (vii) access to healthcare.

**Table 7:** Overview of reported barriers to telemedicine use.

**Reported barriers**	**No. studies (*n*)**	**Studies (%)**
**Technological**		
- Unavailability of infrastructure or equipment	11	20.4%
- Weak/slow internet connection	8	14.8%
- Technical support	7	13.0%
- Technical issues during consultation	2	3.7%
**Regulatory**		
- Telemedicine policies and regulations	5	14.8%
- Unclear reimbursement policies	3	4.6%
- Privacy concerns		
- **Financial**		
- High implementation costs of telemedicine	4	7.4%
**Related to healthcare users**		
- Privacy concerns	8	14.8%
- Acceptability issues	8	14.8%
- Lack of orientation and understanding	9	16.7%
**Related to healthcare providers**		
- Lack of well-trained providers	8	14.8%
- Acceptability issues	4	7.4%
**Quality of care**		
- Inability to conduct physical examination, perform laboratory tests, prescribe medications, or collect samples	12	22.2%
- Healthcare user misunderstanding	3	5.5%
- Low quality of shared images and videos	2	3.7%
- Lack of face-to-face communication	7	13.0%
- Time effort for telemedicine consultation	2	3.7%
**Medicolegal, privacy and confidentiality**		
- Privacy and confidentiality issues	9	16.7%
- Liability issues and unclear medicolegal regulations	5	9.3%
**Access to healthcare**		
- Exclusion of certain populations	2	3.7%
- Lack of widespread availability of telemedicine	2	3.7%

#### Technological Barriers

Some studies (*n* = 11, 20.4%) found that a lack of infrastructure and equipment was a barrier to adoption (6, 7, 29, 35–37, 41, 53–55, 68). Others (*n* = 7, 13%) identified a lack of technical support as a critical impediment to telemedicine solution implementation (6, 27, 36, 37, 46, 48, 61), as well as poor or slow internet connection (*n* = 8, 14.8%) (1, 7, 27, 43, 45, 47, 62, 68), and technological challenges (*n* = 2, 3.7%) when undertaking telemedicine consultations (1, 49).

#### Regulatory Barriers

Policies, or the lack of them, were a reported barrier to applying telemedicine in some studies (*n* = 8, 14.8%) (6, 29, 31, 35, 36, 40, 45, 54). Nine studies (16.7%) reported confidentiality and privacy issues as significant barriers to telemedicine (6, 36, 37, 40, 41, 46, 47, 49, 57). Liability and unclear medicolegal standards were also mentioned as limits of telemedicine's use in other studies (*n* = 5, 9.3%) (6, 41, 46, 47, 49).

#### Financial Barriers

High implementation costs of telemedicine were found a barrier (*n* = 4, 7.4%) (35, 43, 45, 61), as well as nontransparent payment process (*n* = 3, 5.6%).

#### Barriers Related to Healthcare Users

Numerous studies identified healthcare consumers as the main barrier to implementing telemedicine services. Users reported privacy issues in some studies (*n* = 8, 14.8%) (7, 28, 38, 39, 46, 55, 58, 69), as well as a lack of acceptance for telemedicine as a mode of healthcare delivery (*n* = 8, 14.8%) (28, 38, 39, 46, 55, 58, 69). Additionally, several studies (*n* = 9, 16.7%) discovered a deficiency in training and comprehension of healthcare users (7, 28, 33, 38, 39, 46, 55, 58, 69).

#### Barriers Related to Healthcare Providers

A few studies (*n* = 8, 14.8%) indicated that a lack of appropriately qualified healthcare personnel was a barrier to utilizing telemedicine (6, 27, 33, 36, 37, 46, 48, 61), as well as healthcare providers' lack of tolerance and unwillingness to use telemedicine technologies (*n* = 4, 7.4%) (27, 30, 36, 56).

#### Quality of Care

The inability to conduct a physical examination and physically touch the patient, as well as run laboratory tests, administer drugs, or collect samples, was found to potentially not only result in a misdiagnosis but also in lower satisfaction with telemedicine (*n* = 12, 22.2%) (1, 29, 30, 35–37, 46, 48, 49, 53, 54, 56). A few articles (*n* = 3, 5.5%) revealed misdiagnosed cases that could have been related to the physical distance of telemedicine (44, 46, 49). Three studies (5.5%) reported that patient misunderstandings occurred due to communication challenges, and in one case, the patient wrongly took the medication (38, 49, 50). Two studies (3.7%) discovered that the low quality of uploaded photographs and videos may be a telemedicine weakness (24, 27). A few studies (*n* = 7, 13%) revealed that a lack of face-to-face communication between a healthcare professional and a patient could result in the loss of a good relationship and, as a result, have an adverse impact on the quality of care provided (1, 29, 33, 47, 63, 70, 71), as well as telemedicine consultations taking longer to prepare for and conduct than face-to-face consultations (*n* = 2, 3.7%) (36, 37).

#### Access to Healthcare

Telemedicine was found to exclude digitally illiterate people and the elderly who might have limited technical skills (*n* = 2, 3.7%) (57, 63). In addition, the lack of widespread availability of telemedicine was identified as a limitation (*n* = 2, 3.7%) (36, 38).

## Discussion

We found that telemedicine was adopted as a means of healthcare delivery in some studies in order to reduce transmission of COVID-19 while also ensuring continuity of care (32, 47, 48). There were nearly as many publications in the first 4 months of 2021 as in the entire year of 2020, suggesting that interest in telemedicine has surged in LMICs during COVID-19 (see Table 2). The majority of studies focused on middle-income countries, including India, China, Brazil, and Turkey, who adopted telemedicine in response to the COVID-19 pandemic.

### Telemedicine: Technical Characteristics

We found that the infrastructure necessary to implement telemedicine solutions in clinical practice was mostly in place. Mobile phones were extensively available and widely used (72), allowing for the swift implementation of telemedicine services. Some countries have promoted the use of telemedicine solutions by enacting new laws and regulations that relaxed pre-existing restrictions on the use of telecommunications for medical purposes. In the case of Brazil, they passed a bill “for the use of telemedicine on an exceptional basis and as long as the fight against COVID-19 contagion lasts […], authorizing the use of telemedicine in any activities in the health field in Brazil, including teleconsultation, as long as the COVID-19 crisis lasts” (6).

Similarly, in March 2020, both the Ministry of Health and Family Welfare and the Indian Medical Council legalized the use of telecommunications such as text messaging, video, and audio conversations for the purpose of medical consultations and the exchanging of medical information between doctors (30, 66). This was also regulated by the Medical Council of India, which established standards for its use and granted medical practitioners the authority to prescribe pharmaceuticals digitally, except for banned drugs such as narcotics (66). In Lebanon, the Lebanese Order of Physicians and the Ministry of Public Health have urged physicians to utilize telemedicine wherever possible (4).

Synchronous communication, real-time bilateral interaction between patients and healthcare providers via voice calls, video calls, or even instant messaging services, was the most often used way of communication (73). This may best emulate face-to-face interaction and allow for better and more transparent communication between the patient and the healthcare professional, as questions and concerns can be addressed directly, and the healthcare professional receives direct feedback and can ensure that the patient has obtained the necessary information. Only one study used an asynchronous communication mode, email (30). WhatsApp, a commercial instant messaging App, was the most popular platform for telemedicine consultations.

We also found that other commercial apps and software were frequently used, like Zoom and WeChat, but also Facebook (social media) and Apple's FaceTime (videoconferencing). During COVID-19, when social distancing was required, WhatsApp and other commercial apps seemed to offer a simple, readily available platform for consultations among healthcare providers, facilitating telemedicine services and avoiding in-person encounters (74). For example, WhatsApp provides end-to-end encrypted instant messaging, protecting the privacy of the message, and it also seems to be a low-barrier communication tool already widely used in healthcare settings for video calls, phone calls, voice messages, and the sharing of images and videos (74–76).

On the other hand, WhatsApp is a commercial platform, and it is unclear how the users' WhatsApp collected data is processed, handled and stored (77, 78). WhatsApp, amongst others, does not comply with the EU General Data Protection Regulation (GDPR) nor with the United States Health Insurance Portability and Accountability Act (HIPAA) (77). WhatsApp poses a range of challenges, for example, (I) received messages can be deleted, (II) WhatsApp does not keep a record of message exchanges undermining an audit, (III) if a healthcare worker leaves their employer, they still need access to sensitive information—their WhatsApp account would need to be deleted to comply with data privacy and security regulations—, (IV) sensitive information may be sent by accident to other WhatsApp contacts, and (V) it is challenging to avoid giving information in WhatsApp communications that could lead to the patient's identification (77).

Overall, almost all commercial apps and services face similar problems as WhatsApp. Their limitations necessitate alternative software appropriate for the healthcare context, yet, there was a scarcity of such solutions in our review. This begs the question of how to avoid the widespread use of commercial solutions that do not provide the secure environment required to protect the privacy of its users and the data provided. Simply put, the ease of use of these apps will certainly boost their use for professional purposes, which will further exacerbate data protection and privacy challenges. Further research, as well as awareness and initiatives by healthcare professionals, healthcare users, and decision makers, are urgently needed in this area. Solutions are already available, like for example, secure and privacy-compliant messengers like Threema (79) and others.

### Telemedicine Usage in Clinical Practice

Many of the included studies used telemedicine to give medical consultations for diagnosis or follow-up, specialist consultations, health information exchanges between healthcare providers, laboratory testing, drug prescription, and drug delivery. In addition, telemedicine was utilized to triage healthcare users in pandemic and crisis situations, for remote screening, mainly to reduce unnecessary exposures during the COVID-19 pandemic, and to provide counseling to healthcare users.

Telemedicine services were facilitated as part of the digital health ecosystems, such as digital health records and online and virtual clinics. This guarantees that patients continue to receive clinical care, reducing physical crowding of patients in hospital facilities (6). Telemedicine has been reported as a tool for securely storing healthcare users' health information as a digital health record in a secure location and promoting easy information exchange among healthcare practitioners (37).

Although beneficial for exchanging health information, concerns about patient privacy and confidentiality were mentioned in included studies of this review, as some healthcare users have expressed worry about data protection and privacy (1). Data privacy and its safe management are crucial features of telemedicine that must be considered when deploying digital health solutions as part of the healthcare environment, including telemedicine implementations. Only if all engaged actors have a sufficient degree of digital literacy and awareness about digital health technologies like telemedicine, as is true for many other applications like electronic medical education (80), such systems can be used to their maximum potential and for the intended purpose. This will almost certainly require infrastructure modifications and changes in routines, as digital health applications raise the need for tight collaboration with IT to support their expertise in the setup, maintenance, and training of such systems.

### Reported Needs for Employing Telemedicine, Particularly During the COVID-19 Pandemic

Many included studies have emphasized telemedicine's need for preventing disruptions in healthcare services, particularly non-urgent healthcare, and in continuing healthcare delivery while conforming to COVID-19-related restrictions and lockdown measures that may impede access to healthcare. Additionally, the included studies voiced a need for telemedicine to ensure that persons suffering from chronic conditions that require continuity of care receive critical medical treatment and counseling while simultaneously protecting them from infection risks that may be much higher in healthcare facilities. Telemedicine may also serve as a safeguard for healthcare users and providers since it may prevent close face-to-face contact with individuals who are COVID-19 positive but are asymptomatic or unaware of their infection. Particularly given that healthcare facilities in LMICs are frequently overcrowded, telemedicine may alleviate overcrowding in healthcare facilities, hence reducing the risk of infection and alleviating part of the stress on these healthcare facilities (26).

The COVID-19 pandemic has increased demand for infection control equipment, such as protective face masks; included studies stated the necessity for telemedicine to prevent hospital admissions in specific cases and its successfully usage. Therefore, telemedicine could be a long-term option to deal with diverse shortages. Another identified need that encouraged the implementation of telemedicine was the scarcity of doctors, still a challenge in many LMICs. According to Bhatia et al. (38), India's doctor-to-patient ratio is 0.77:1,000, which is lower than WHO recommendations and may make healthcare unavailable to a large number of people. Telemedicine has the potential to be the instrument that connects the healthcare user and the healthcare provider regardless of geographical boundaries or the local availability of healthcare providers (64).

### Reported Facilitators and Barriers of Employing Telemedicine, Particularly During the COVID-19 Pandemic

Many healthcare users reported increased access to healthcare because of telemedicine. Due to lockdowns and restrictions that at times only allowed urgent/acute COVID-19 cases to seek medical attention in healthcare facilities, healthcare consumers were turning to telemedicine as an alternative to continue receiving medical attention (36). Healthcare users also reported saving money directly if providers offered online consultations at a lower cost than face-to-face, and indirectly by saving money on transportation and possibly lodging if the healthcare institution was far away. Also mentioned were time savings, as it was unnecessary to travel long distances to access the healthcare facility (27, 41, 54).

Overall, healthcare users' attitudes toward telemedicine were quite positive. In some cases, family members and caregivers assisted in telemedicine consultations by offering technical assistance or using their mobile phones to conduct the session. Nevertheless, acceptance of telemedicine by healthcare users was identified as a critical barrier, mainly due to privacy and confidentiality, a lack of incentive to use telemedicine, or a lack of telemedicine orientation and comprehension. Several studies have found that these issues were more widespread in older populations and in people with lower educational levels. Any large-scale adoption and implementation of telemedicine services needs to find ways of making such services accessible to the technologically illiterate and the elderly, who had less total technological exposure. Furthermore, it should be investigated how the doctor-patient relationship can be maintained when using telemedicine, as well as how to avoid missing “warmth,” which may have a negative impact on patient compliance and the quality of care (29).

Like healthcare users, healthcare providers were also able to save time and money by using telemedicine. For example, healthcare providers, who have been infected or quarantined, can continue to deliver medical consultations without risking their own or others' health (60). Telemedicine was also reported to be used to supplement much-needed PPE, which was in short supply during the COVID-19 pandemic. Overall, we found that healthcare providers were quite pleased with the use of telemedicine, most likely due to time and cost savings, but also to a considerably decreased risk of infection linked with fewer face-to-face consultations with potentially infected healthcare users.

Also, healthcare providers have received at times specific training on how to use telemedicine platforms and the protocols that must be followed when conducting a telemedicine consultation. Implementing telemedicine on a larger scale will likely result in less overcrowding in healthcare facilities, as included studies have also reported. Aside from the COVID-19 pandemic, telemedicine's use may improve the quality of healthcare systems by permitting the creation of digital health records that can be securely stored on the cloud. This health information can then be quickly and efficiently exchanged between healthcare users and providers. However, healthcare provider acceptance of telemedicine may reduce the likelihood of adoption, which is exacerbated by a lack of training on how to use telemedicine platforms and a lack of protective rules and payment policies.

On the other hand, a lack of available infrastructure and/or equipment needed to execute telemedicine solutions was reported. This lack of availability was more noticeable in rural areas than in urban and metropolitan areas (36). Furthermore, the poor/slow internet connection in some LMICs, particularly in rural regions, may be a barrier to the adoption of telemedicine. According to our findings, a key impediment to introducing telemedicine solutions was a lack of technical support. Telemedicine, particularly when building a new platform, necessitates a constant process of technical support and development (7, 36, 37). Otto and Harst found that an effective telemedicine application requires a multi-level approach that includes 11 key factors: patient, healthcare provider, culture, and disease (people-related); health sector, standards/guidelines, legal framework, finance, organization, and methodology (process-related); and technology (object-related) (81). Thus, before implementing telemedicine infrastructure, it should be ensured that prerequisites are met. Although we have seen in the included studies that urgency can act as a catalyst pushing beyond reported barriers, as the need of the situation appears to make people more accepting of what works.

Furthermore, the issue is that in many LMICs, the rules, regulations, and policies, or lack thereof, may impede the adoption and utilization of telemedicine platforms (35, 40, 51). Often, there are no clear regulations in place, which may discourage stakeholders, and potentially lead to the medical community's opposition to adopting telemedicine solutions (4). In addition, in some included studies, healthcare providers were hesitant to use telemedicine solutions due to the lack of clarity around reimbursement procedures (4).

Trust is an essential component for telemedicine's use for both sides, healthcare users and providers, and should be investigated further. There are several trust models available for implementation; for example, Nundy et al. (82) proposed a three-component model comprised of competency (physicians' clinical mastery, patients' knowledge and self-efficacy regarding their own health), motive (patients' trust in physicians to act solely in their best interests), and transparency (understanding of clinical decisions). Overall, it is vital to ensure that there are no negative consequences on the quality of care when telemedicine is used in healthcare. Otherwise, it will undermine trust and increase the risk of digital health solutions, such as telemedicine, being rejected. Of course, patient data security is critical, especially as cyberattacks become more sophisticated (83). It appears essential to consider introducing digital health training into the medical curriculum to develop capabilities, improve skills, boost transparency, and invest in patient information to improve transparency (84, 85).

## Conclusion

Main reasons for introducing telemedicine in LMICs during COVID-19 were to maintain non-emergency healthcare, enhance access to healthcare providers, and reduce the risk of infection among health users and providers. Special legislation has also been developed in several countries, including Brazil, Lebanon, and India, to facilitate the introduction and implementation of telemedicine services to counterbalance the impacts of COVID-19 on the healthcare sector. Overall, healthcare providers and users have shown a high level of acceptance for telemedicine services, and in most included studies, telemedicine was able to improve access to healthcare. Our scoping review is, to the best of our knowledge, the first to map all known information focusing on LMICs on a broad scale, incorporating lessons learned from the COVID-19 pandemic.

However, there are still infrastructural and regulatory barriers that could impede widespread use of telemedicine, even though it offers a variety of benefits such as reduced face-to-face interaction, prevention of infectious disease spread, de-congestion of healthcare facilities, access to healthcare and specialists even in rural areas, and a time and cost-saving component. It is vital to ensure that telemedicine does not jeopardize the quality of care provided. Otherwise, confidence will deteriorate, and digital health solutions such as telemedicine may be rejected. Patient data security is vital, significantly as threats increase. Healthcare providers should prioritize protecting sensitive data by avoiding the usage of commercial apps and services such as WhatsApp, Zoom, WeChat, and others. The medical curriculum may consider incorporating digital health training to promote capacity, skills, and transparency, which may improve patients' and health care providers' ability to harness such technologies. As a result, we recommend that regulatory bodies revise and increase adoption of telemedicine services as the results demonstrate their efficacy. Also, more research is needed, particularly in low-resource contexts, to better understand the barriers and facilitators in such contexts, as they may differ in terms of internet access, electricity, access, and acceptance of technology, amongst other things.

## Limitations

The identified limitations in this review were the exclusion of all articles published in any language other than English and only searching one database for grey literature (Google Scholar). This may increase the risk of selection biases in this research. Since only one of the studies included was conducted in a low-income country, the transferability and generalizability of our findings must be examined in each context.

Our search included all countries that were defined as low-income- (LICs), lower-middle-income- (LMICs), or upper-middle-income- (UMICs) countries by the World Bank in May 2021; however, our search primarily yielded results in North Africa and very limited only covering South Africa or other regions in Africa, as well as Asia and South America, which may limit the applicability of our findings to all LMIC. This may indicate a lack of research or publications or also telemedicine utilization as searches did not yield more results from these countries.

## Data Availability Statement

The original contributions presented in the study are included in the article/Supplementary Material, further inquiries can be directed to the corresponding author.

## Author Contributions

The objective and research question were developed by KM and SB. The first round of data screening was conducted by KM and CJ. The second round was conducted by KM. Data extraction, analysis, and interpretation were conducted predominantly by KM with support from CJ and SB. KM wrote the manuscript draft. SB revised the full manuscript. SB and CJ all contributed to further manuscript revision. The final version of the manuscript was approved by all authors.

## Funding

This work was supported by the Else Kröner-Fresenius-Stiftung (2019_HA25). The funders were not involved in study design, collection, management, analysis or interpretation of data, neither in the writing of this report or in any decision to submit this report for publication. For the publication fee we acknowledge financial support by Deutsche Forschungsgemeinschaft within the funding programme “Open Access Publikationskosten” as well as by Heidelberg University.

## Conflict of Interest

The authors declare that the research was conducted in the absence of any commercial or financial relationships that could be construed as a potential conflict of interest.

## Publisher's Note

All claims expressed in this article are solely those of the authors and do not necessarily represent those of their affiliated organizations, or those of the publisher, the editors and the reviewers. Any product that may be evaluated in this article, or claim that may be made by its manufacturer, is not guaranteed or endorsed by the publisher.
