# Supplementary material for: Telemedicine in Low- and Middle-Income Countries During the COVID-19 Pandemic: A Scoping Review
Source: Front Public Health. 2022 Jun 22;10:914423. doi: 10.3389/fpubh.2022.914423 (PMC9257012; doi:10.3389/fpubh.2022.914423)
Supplement: Supplementary file 1 [file Data_Sheet_1.docx]

Supplementary Material

# Supplementary Material 1: Search concepts with MeSH terms and keywords used to in PubMed

| **Concept** | **No.** | **Search string** |
| --- | --- | --- |
| Telemedicine | 1 | (((((((telemedicine[MeSH Terms]) OR ("telemedicine"[MeSH Terms] OR "telemedicine/history"[MeSH TeSH Terms] OR "telemedicine/organization and administration"[MeSH Terms] OR "telemedicine/trends"[MeSH Terms])) OR (telehealth[MeSH Terms])) OR (consultation, remote[MeSH Terms])) OR (telepathology[MeSH Terms])) OR (teleradiology[MeSH Terms])) OR ("telerehabilitation"[MeSH Terms])) |
|  | 2 | (((("mobile health"[All Fields]) OR ("telehealth"[All Fields])) OR ("telemedicine"[All Fields])) OR ("ehealth"[All Fields])) OR ("mhealth"[All Fields]) |
| COVID-19 | 3 | ("coronavirus"[MeSH Terms] OR "coronavirus 229e, human"[MeSH Terms] OR "coronavirus infections"[MeSH Terms] OR "coronavirus/analysis"[MeSH Terms] OR "coronavirus infections/blood"[MeSH Terms] OR "coronavirus infections/immunology"[MeSH Terms]) AND ("covid 19"[MeSH Terms] OR "covid 19 testing/diagnosis"[MeSH Terms] OR "covid 19 testing"[MeSH Terms] OR "covid 19 serological testing/trends"[MeSH Terms] OR "covid 19/diagnosis"[MeSH Terms]) |
|  | 4 | ((((("corona"[All Fields]) OR ("covid"[All Fields] OR "covid 19"[All Fields])) ) OR ("coronavirus"[All Fields] OR "coronavirus 19 covid 19"[All Fields] OR "coronavirus 19 disease"[All Fields] OR "coronavirus 19 disease covid"[All Fields] OR "coronavirus 19 diseases covid"[All Fields] OR "coronavirus 19 disease covid 19"[All Fields] OR "coronavirus 19 diseases"[All Fields] OR "coronavirus 19 diseases covid 19"[All Fields] OR "coronavirus 2"[All Fields])) OR ("novel corona"[All Fields] OR "novel corona virus"[All Fields] OR "novel corona virus 19"[All Fields] OR "novel corona virus 2019"[All Fields] OR "novel corona virus 2019 ncov"[All Fields] OR "novel corona virus disease"[All Fields] OR  "novel corona virus disease 2019"[All Fields] OR "novel corona virus infection"[All Fields] OR "novel corona virus sars"[All Fields] OR "novel corona virus sars cov 2"[All Fields] OR "novel corona virus sars cov"[All Fields] OR "novel coronavirus 2019"[All Fields] OR "novel coronavirus 2"[All Fields] OR "novel coronavirus"[All Fields] OR "novel coronavirus 19"[All Fields] OR "novel coronavirus 2019 covid"[All Fields] OR "novel coronavirus 2019 covid 19"[All Fields] OR "novel coronavirus 2019 ncov"[All Fields] OR "novel coronavirus 2019 disease"[All Fields] OR "novel coronavirus 2019 infection"[All Fields] OR "novel coronavirus disease 2019"[All Fields])) OR ("covid 19"[All Fields]) |
| Low- and Middle- Income Countries | 5 | ("developing countries"[MeSH countries/classification"[MeSH countries/economics"[MeSH countries/history"[MeSH countries/statistics and numerical data"[MeSH Terms] OR "developing countries/organization and administration"[MeSH Terms]  Terms] OR Terms] OR  "developing "developing "developing "developing  Terms] OR Terms] OR |
|  | 6 | (((("lmic"[All Fields] OR "lmic africa"[All Fields] OR "lmic context"[All Fields] OR "lmic contexts"[All Fields] OR "lmic countries"[All Fields] OR "lmic health"[All Fields] OR "lmic health care"[All Fields] OR "lmic health systems"[All Fields] OR "lmic health system"[All Fields] OR "lmic innovations"[All Fields] OR "lmic low and middle income countries"[All Fields] OR "lmic low and middle income country"[All Fields]) OR ("low income"[All Fields] OR "low income contexts"[All Fields] OR "low income countries"[All Fields])) OR ("low income countries"[All Fields] OR "low income countries and lower middle"[All Fields] OR "low income countries and lower middle income"[All Fields] OR "low income countries and lower middle income countries"[All Fields] OR "low income countries health"[All Fields] OR "low income countries health systems"[All Fields] OR "low income country context"[All Fields] OR "low income country contexts"[All Fields])) OR ("middle income countries"[All Fields] OR "middle income countries data"[All Fields] OR "middle income countries guidelines"[All Fields] OR "middle income countries health"[All Fields] OR "middle income countries health systems"[All Fields] OR "middle income countries iconic"[All Fields] OR "middle income countries iconic africa"[All Fields] OR "middle income countries low income"[All Fields] OR "middle income countries lmic s"[All Fields] OR "middle income countries settings"[All Fields] OR "middle income countries mic"[All Fields] OR "middle income countries the"[All Fields] OR "middle income country settings"[All Fields])) OR ("developing countries"[All Fields] OR "developing countries 1"[All Fields] OR "developing countries and clinical"[All Fields] OR "developing countries economics"[All Fields] OR "developing countries many"[All Fields]) OR "low income countr*" OR "middle income countr*" OR Afghanistan OR "Guinea-Bissau" OR "Sierra Leone" OR "Burkina Faso" OR Haiti OR Somalia OR Burundi OR "North Korea" OR "Democratic People's Republic of Korea" OR "South Sudan" OR "Central African Republic" OR Liberia OR Sudan OR Chad OR Madagascar OR "Syrian Arab Republic" OR "Syria" OR "Democratic Republic of the Congo" OR "Congo-Kinshasa" OR Malawi OR Tajikistan OR Eritrea OR Mali OR Togo OR Ethiopia OR Mozambique OR Uganda OR "The Gambia" OR Niger OR Yemen OR Guinea OR Rwanda OR Angola OR Honduras OR "Papua New Guinea" OR Algeria OR India OR Philippines OR Bangladesh OR Kenya OR "São Tomé and Principe" OR Benin OR Kiribati OR Senegal OR Bhutan OR "Kyrgyz Republic" OR Kyrgyzstan OR "Solomon Islands" OR Bolivia OR "Laos" OR "Lao People's Democratic Republic" OR "Sri Lanka" OR "Cabo Verde" OR Lesotho OR Tanzania OR Cambodia OR Mauritania OR "Timor-Leste" OR "East Timor" OR Cameroon OR "Micronesia" OR "Federated States of Micronesia" OR Tunisia OR Comoros OR Moldova OR Ukraine OR "Republic of the Congo" OR "Congo-Brazzaville" OR Mongolia OR Uzbekistan OR "Côte d'Ivoire" OR "Ivory Coast" OR Morocco OR Vanuatu OR Djibouti OR Myanmar OR Burma OR Vietnam OR Egypt OR Nepal OR "West Bank and Gaza" OR "El Salvador" OR Nicaragua OR Zambia OR Eswatini OR Nigeria OR Zimbabwe OR Ghana OR Pakistan OR Albania OR Fiji OR Montenegro OR "American Samoa" OR Gabon OR Namibia OR Argentina OR Georgia OR "North Macedonia" OR Armenia OR Grenada OR Paraguay OR Azerbaijan OR Guatemala OR Peru OR Belarus OR Guyana OR "Russian Federation" OR "Russia" OR Belize OR Indonesia OR Samoa OR "Bosnia and Herzegovina" OR Iran OR "Islamic Republic of Iran" OR Serbia OR Botswana OR Iraq OR "South Africa" OR Brazil OR Jamaica OR "Saint Lucia" OR Bulgaria OR Jordan OR "Saint Vincent and the Grenadines" OR "Saint Vincent" OR China OR Kazakhstan OR Suriname OR Colombia OR Kosovo OR Thailand OR "Costa Rica" OR Lebanon OR Tonga OR Cuba OR Libya OR Turkey OR Dominica OR Malaysia OR Turkmenistan OR "Dominican Republic" OR Maldives OR Tuvalu OR "Equatorial Guinea" OR "Marshall Islands" OR "Venezuela" OR "Bolivarian Republic of Venezuela" OR Ecuador OR Mexico |

# Supplementary Material 2: Data extraction template

| Title |
| --- |
| Author |
| Year |
| DOI |
| Language |
| Full text URL |
| Country in which the study conducted World Bank classification District/Region |
| Urban/Rural |
| Objective/Purpose of study |
| Study design |
| Study type Platform used |
| Synchronous/Asynchronous |
| Mode of communication (telephone, voice video, text message, email) |
| Telemedicine platform Implementation Date (duration) |
| Target groups |
| healthcare institute (Single physician/clinic/hospital) |
| Meidcal Speciality |
| Perceived need for Telemedicine solutions Perceived Benefits of using telemedicine Applications |
| Facilitators |
| Barriers |
| Shortcomings |
| Study perspective (User-patient, health worker, provider, state) |
| Instrument used for measurement (interview/survey...) |
| Sample (key characteristics e.g age, socio economic status) |
| Sample size |
| Sampling approach Results |
| Conclusions |
| Recommendations Methodological issues identified Notes |
